# Tai Chi for Lower Urinary Tract Symptoms and Quality of Life in Elderly Patients with Benign Prostate Hypertrophy: A Randomized Controlled Trial

**DOI:** 10.1155/2012/624692

**Published:** 2011-10-03

**Authors:** Seil Jung, Eun-Nam Lee, Sook-Ryon Lee, Mi-Sook Kim, Myeong Soo Lee

**Affiliations:** ^1^Department of Urology, College of Medicine, Dong-A University, Busan, Republic of Korea; ^2^Department of Nursing, Dong-A University, Busan, Republic of Korea; ^3^Department of Nursing, Masan University, Chang Won, Republic of Korea; ^4^Salus Cancer Rehabilitation Exercise Institute, Busan, Republic of Korea; ^5^Brain Disease Research Center, Korea Institute of Oriental Medicine, Daejeon, Republic of Korea

## Abstract

Tai chi exercise has been recommended as suitable for the improvement of health in the elderly. The purpose of this study was to investigate the effects of tai chi on lower urinary tract symptoms (LUTSs), quality of life (QoL), and sex hormone levels in patients with benign prostate hypertrophy (BPH). The elderly patients with BPH were randomized to receive tai chi or usual care. Fifty-six participants were randomized into either the tai chi group (*n* = 28) or the control group (*n* = 28). After 12 weeks of treatment, the tai chi group showed significant improvement in LUTS and QoL. There was a significant effect of tai chi on testosterone but no significant effect on insulin or glucose. No serious adverse events were observed during the study period. In conclusion, our results suggest that 12 weeks of tai chi may improve LUTS and QoL in elderly patients with BPH.

## 1. Introduction

With the recent increase in the elderly population and their increased demand for better health-related quality of life (QoL), lower urinary tract symptoms (LUTSs), which produce discomfort in elderly men, are receiving more attention. Benign prostate hypertrophy (BPH), a major cause of LUTS in elderly men, is a urinary tract disease caused by a combination of prostate hypertrophy, lower urinary tract obstruction, and dysfunction of the bladder muscles. Among elderly men over 65 years, the prevalence of BPH increases to 40–70%, and among elderly men over 70 years, it increases to 90% [1]. It is a major health issue among elderly men. 

It is known that those with LUTS experience interference with daily activities, including discomfort, restricted travel and outings, lowered QoL due to concerns about urinary function, prostate cancer, embarrassment about urinary problems, and even psychological problems [2, 3]. 

The causes of BPH have not yet been clarified, but genetic factors, nutrition, lack of exercise, racial differences, and chronic illnesses, such as high blood pressure and diabetes, are believed to play a role. From 60% to 70% of men over 60 have high blood pressure, and over 50% of high blood pressure patients show abnormal histological findings or symptoms of prostate hypertrophy [4]. These symptoms increase with age, showing that hyperactivity of the autonomic nervous system and hyperinsulinemia play important roles in the exacerbation of LUTS due to BPH [5, 6]. Also, increased physical activity has a negative correlation with LUTS and BPH symptoms [7, 8]. Based on these findings, researchers have recommended adequate exercise for patients with BPH because light physical activities will reduce insulin resistance and the activity of the autonomic nervous system. However, the type, time, and intensity of exercise best suited for the alleviation of LUTS due to BPH have not yet been studied. 

Tai chi has been recommended as an exercise suitable for improving elderly men's cardiopulmonary function and muscle strength and for reducing tension, anxiety, and mood disorders [9]. Tai chi has drawn attention because it is easy to learn, inexpensive and can be performed anywhere. Lu and Kuo have reported that tai chi is effective in improving autonomic nervous system-related diseases in the elderly by promoting the activity of the parasympathetic nervous system and reducing the excitement of the sympathetic nervous system [10, 11]. We hypothesized that tai chi would improve LUTS and BPH by modulating the autonomic nervous system. 

The purpose of this study was to investigate whether tai chi improves LUTS, quality of life, and sex hormone levels in patient with BPH. Positive results might lead to tai chi being adopted as a nursing intervention for patients with BPH. 

## 2. Participants and Methods

### 2.1. Patients

**Figure 1:**
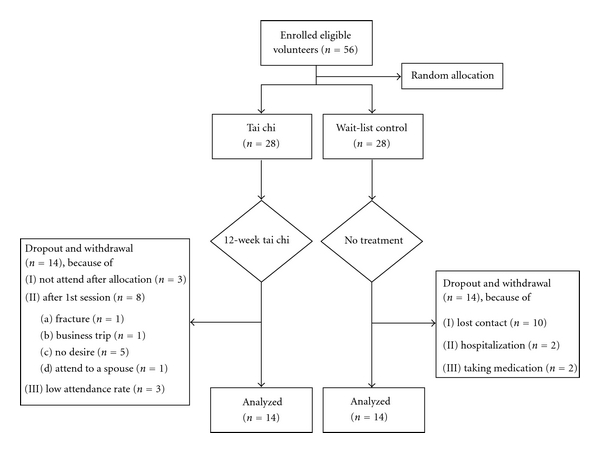
A diagram of the study design, showing the flow of participants.

Patients with BPH were recruited through bulletin board invitations to participate in a 12-week tai chi program at the Dong-A University Medical Center. Patients were eligible to participate in the program if they met the following conditions: (a) male aged over 60 with BPH, (b) able to understand the content of questionnaires and experimental schedules, (c) had not participated in regular exercise in the previous six months, (d) a lower urinary tract symptoms score greater than 25 points, (e) had not received transurethral resection, and (f) were not participating in any other form of CAM. The subjects were informed about the nature of BHP and the study procedures. We received approval for the study from the Dong-A University Hospital's Institutional Review Board before we approached the subjects; all the subjects provided written informed consent (Figure 1). 

### 2.2. Randomization

We randomly assigned the patients to either the tai chi group or the control group by tossing a coin. There was no blinding or allocation concealment for either the participants or the practitioner. Nursing assistants who did not participate in the trial and who were blinded to the allocation results performed the outcome assessments.

### 2.3. Outcome Measures

This study's outcome measures included the following: (1) the international prostate symptom score (IPSS), (2) urination-related quality of life, (3) serum testosterone, and (4) blood glucose and insulin. The outcome measures were assessed before and 12 weeks after the intervention by a nurse who did not know the experimental protocol or the subjects' allocation.

#### 2.3.1. Lower Urinary Tract Symptoms (LUTSs)

The LUTSs were measured using the IPSS. The IPSS is identical to the American Urology Association (AUA) symptom index, which was developed by AUA in 1991. The WHO international consultation on BPH changed its name to IPSS [12]. It consists of 7 items: 3 items for storage symptoms and 4 items for voiding symptoms. For each item, “not at all” is scored as 0, “1 in 5 times” is scored as 1, “1 in 3 times” is scored as 2, “1 in 2 times” is scored as 3, “2 in 3 times” is scored as 4, and “almost always” is scored as 5. Total scores of 0 to 7, 8 to 19, and 20 to 35 indicate mild, moderate, and severe symptoms, respectively. The IPSS has been translated into many languages and is widely used around the world. Choi et al. [13] created a Korean version and verified its validity and reliability. In their study, the reliability, as measured by Cronbach's *α*, was  .91.

#### 2.3.2. Urination-Related  Quality  of  Life  (QoL)

The urina-tion-related QoL questionnaire consisted of 18 items in 3 subscales that used a 5-point Likert response scale [14]. The three subscales were discomfort, worry, and interference with daily activities.

**Discomfort**

For the degree of discomfort, 7 international prostrate symptoms were measured. The subjective discomfort felt by each respondent was quantified. The 5-point scale ranged from 0 (no symptoms) to 4 (very uncomfortable). The reliability, as measured by Cronbach's *α*, was  .945 in the present study.

**Worry**

A 5-point Likert scale consisting of 4 questions that was developed by Epstein et al. [14] was used to measure the degree of worry due to LUTS. The questions concerned voiding function, sexual function, and worry about the possibility of getting prostate cancer. The scores ranged from 0 (not at all worried) to 4 (very much worried). In a study by Kim [15] using this scale, the reliability was  .89. The reliability, as measured by Cronbach's *α*, was  .87 in the present study.

**Interference with Daily Activities**

A 5-point Likert scale consisting of 7 questions that was developed by Epstein et al. [14] was used to measure the degree of interference with daily activities due to LUTS. The questions concerned interference with beverage intake, social life, sleep, driving, going to an unfamiliar place, and outdoor exercise due to LUTS over a one-month period. The degree of interference ranged from 0 (not at all) to 4 (very much). In Epstein et al.'s study [14], the internal consistence was  .81, and the reliability, as measured by Cronbach's *α*, was  .84 in the present study.

#### 2.3.3. Testosterone

Serum testosterone was measured using a radioimmunoassay (Coat-A-Count Total Testosterone Kit, DPC, LA, USA). The units for serum testosterone were ng/mL. The normal value for males over 50 years old is 1.81*∼*7.58 ng/mL. 

#### 2.3.4. Insulin Resistance

Insulin resistance is the reduced response of peripheral tissues to insulin action. Methods to evaluate it based on fasting serum insulin and fasting blood glucose have been developed. One of these is the quantitative insulin sensitivity check index (QUICKI). This index is a measure of insulin sensitivity rather than insulin resistance (i.e., the lower the QUICKI score, the higher the insulin resistance). QUICKI is recommended for measuring insulin resistance because it has relatively small measurement error and is well correlated with clamp methods, which are the standard test [16]. Blood was collected from the medial cubital vein after 8 hours of fasting. The fasting glucose level was measured in mg/dL using the glucose oxidation method (747 autoanalysis, Hitachi, Tokyo, Japan). After measuring the serum insulin in *μ*U/mL using a radioimmunoassay, the QUICKI value was calculated using the formula 1/log fasting insulin + log fasting blood glucose.

### 2.4. Intervention

The intervention program used 11 basic and 9 combined movements (20 total) that were developed by Dr. Paul Lam. The tai chi program consisted of a warm-up exercise (15 min), 20 main movements (40 min), and a cool-down exercise (5 min). The study's warm-up and cool-down exercises involved stretching and relaxing the head, neck, upper body, lower body, and entire body.

The 11 basic tai chi movements involved the commencement form, opening and closing hands, waving hands in the cloud (left), opening and closing hands, the fair lady working at the shuttle, opening and closing hands, toe kicks left and right, opening and closing hands, waving hands in the cloud (right), opening and closing hands, and the closing form. The nine combined forms included waving hands in the cloud (left), opening and closing hands, stroking the bird's tail (left), opening and closing hands, stroking the bird's tail (right), opening and closing hands, waving hands in the cloud (right), opening and closing hands, and the closing form. When the participants performed the opening and closing hands, they also performed breathing exercises.

The participants in the tai chi group attended classes 3 times weekly for twelve weeks; the classes were led by two tai chi instructors. We individually instructed the participants in the appropriate movements. The patients learned and practiced the motions during the first 5 weeks. The participants were actually performing the routine competently during the last seven weeks. A special guide book for home practice, containing pictures and written descriptions of the exercises in the tai chi program, was produced. The participants were asked to practice their exercises at home using the guide book two times daily (in the morning and evening). The participants recorded the frequency and duration of their home tai chi in their exercise logs, which the instructors assessed during every weekly session. A videotape was available if the participants desired.

### 2.5. Procedure

The subjects in both groups were diagnosed as BPH and did not receive standard drug therapies. The control subjects received no other treatment and did not participate in any structured exercise programs during the study period. They were contacted by the researchers twice weekly by telephone to confirm that they were not taking part in any other exercise activities and to provide an impetus to keep them participating in the study. The control group subjects who were interested in tai chi were provided with an exercise program after the study ended.

### 2.6. Sample Size

We wished to estimate the sample size that would be sufficient to detect an appropriate difference in the IPSS between the tai chi and control groups. Because no previous trials have been performed, we calculated the sample size using the results of a drug trial. In this drug trial, the mean difference in the IPSS between the groups was −3.9 [17]. We also used the pooled standard deviation between the groups from this study (4.6). The alpha value and desired power were 0.05 and 0.8, respectively. From these values and an assumed 20% dropout rate, we calculated a required sample size of 28 for each group in an independent, two-sample *t-test* using the following equation:

(1) $$n=\frac{2{\left(Z\left(\alpha /2\right)+Z\left(\beta /2\right)\right)}^{2}\sigma^{2}}{{\left(\mu_{c}-\mu_{t}\right)}^{2}}.$$

### 2.7. Statistical Analysis

The results were analyzed using SPSS software. All the outcomes were compared using the unpaired *T-test* for the between-group comparisons and the paired *T-test* for the within-group comparisons between baseline and posttreatment values.

## 3. Results

Fifty-six patients were eligible under our study's criteria. We randomly allocated these patients to either the tai chi group (*n* = 28) or the control group (*n* = 28) by tossing a coin. The dropout rates were 50% in both groups. Both pre- and posttest data were available from 14 subjects in the tai chi group and 14 controls. In the tai chi group, 3 did not attend after enrollment, and 8 patients dropped out after the first session for the following reasons: not desiring to attend further sessions (*n* = 5); fractures (*n* = 1); the need to attend to a spouse (*n* = 1); a business trip (*n* = 1). We were unable to use the data from 3 patients due to their low attendance rates. The primary reasons for dropping out in the control group were failure to contact (*n* = 10), medication (*n* = 2), and a further examination for possibility of cancer (*n* = 2). We conducted an additional analysis to compare the demographic and pretest data of the dropouts to those of the remaining members of the tai chi and control groups and found no significant differences.

Table 1 shows the demographic characteristics of subjects in the tai chi and control groups. The groups did not differ significantly by age, educational level, perceived health status, surgical history for the prostate, medication, or participation in regular exercise.

As shown in Table 2, there were no significant differences between the two group in the IPSS, QoL scores, urination-related discomfort, worry and concern, and interference with daily activities subscales, QUICKI, or serum levels, including testosterone, insulin, and blood glucose.

Table 3 shows the results for the outcome measures. After 12 weeks of treatment, the IPSS differed significantly between the tai chi group and the control group (*t* = −2.36, *P* = 0.03). For the total QoL score, the change was more significant in the tai chi group than in the control group (*t* = −3.06, *P* = 0.005). Regarding the QoL worry & concern subscale, there was significant improvement in the tai chi group compared with the control, while the results failed to show significant improvements in the urination-related discomfort or interference with daily activity subscales.

The level of testosterone increased significantly in the tai chi group compared to the control, while there were no significant intergroup differences in insulin, blood glucose, or the QUICKI.

No adverse effects associated with the practice of tai chi were reported by the participants.

## 4. Discussion

This randomized clinical trial investigated the efficacy of 12 weeks of tai chi on LUTS, quality of life, and sex hormone levels in elderly patients with BPH. Our study showed that tai chi significantly improves LUTS compared to a control group. The total of quality of life score was significantly more improved in the tai chi group than in the control group after 12 weeks. The worry and concern QoL subscale at 12 weeks showed a statistically significant difference from the control group. The testosterone showed significant intergroup differences, suggesting greater modulation of hormonal effects in relation to LUTS in the tai chi group compared to the control. This finding has not been previously reported.

This trial found up to a 31% and 24% within-group improvement from baseline for the LUTS and QoL scores, respectively, in the tai chi group. These results suggest a promising role for tai chi in LUTS related to BPH. These results are consistent with previous reports that increased physical activity and exercise are consistently related to a decreased risk for BPH and LUTS [4, 8]. There is also a meta-analysis showing that physical activity is associated with a 25% decreased risk of BPH and LUTS compared to a sedentary life style [7]. Although there have been no previous attempts to test exercise for treating BPH and LUTS, our study is strongly consistent with the beneficial effects of exercise for BPH and LUTS. 

We recalculated the power of our trial for the mean IPSS differences using the two-sample *t*-test with unequal variances. The total power of our trial was 60%, in spite of the prior sample size calculation. The recalculated sample size was 22 for each group, without considering participants who dropped out or withdrew. The decreased power from the original sample calculation was caused by high dropout and withdrawal rates (50%), which resulted from the age of the participants and the lack of proper compliance management.

Assuming that tai chi is a potentially useful treatment option for patients with BPH, its possible mechanisms of action may be of interest. When performed regularly, the physical exercise of tai chi affects the cardiovascular and metabolic processes [10, 18]. It has been reported that lifestyle modifications are beneficial for reducing the risk of developing BPH and LUTS [19–22]. Considering these findings, tai chi may prevent BPH and LUTS. However, no trials have tested the efficacy of exercise for treating BPH and LUTS. The possible mechanisms of improved LUTS in BPH include a decreased resting sympathetic tone in the prostate, an alteration in the levels of certain hormones (insulin and testosterone), and a reduction in prostatic inflammation through decreased oxidative damage. However, our study failed to show any effect of tai chi in modulating insulin metabolism compared to the control group. None of these hypotheses were proven, and the true mechanisms for the effect of tai chi on LUTS and BPH need to be elucidated by further research. 

The limitations of this study include the relatively short period of observation (less than 6 months) and the high dropout and withdrawal rates. Although no significant baseline imbalances in the patient characteristics and LUTS scores were found, this high attrition may have increased the risk of bias because not all of the randomized patients were analyzed. Another risk of bias was from not employing allocation concealment and blinding, although we did use assessor blinding. This factor may have exaggerated the real effects of tai chi for LUTS and other outcomes. We also could not completely control for the placebo or expectation effects due to the lack of an equivalent exercise control group. One of authors participated in conducting the tai chi sessions. Thus, the practitioners' attitudes toward the tai chi group (if present) may have affected the treatment procedure and outcomes, although we attempted to minimize these effects by the extensively controlled research setting; we found no evidence of protocol violations. The number of participants in this study was small, which increased the chance of type II error. Furthermore, we did not use the sample size calculations, and we could not exclude the possibility that the study was underpowered for detecting a meaningful effect of tai chi on several outcome measures. 

## 5. Conclusion

Our results suggest that 12 weeks of tai chi may improve LUTS and QoL in elderly patients with BPH. Future tai chi RCTs should properly address the limitations and difficulties encountered in this study by employing an equivalent control group. 

## Figures and Tables

**Table 1:** The demographic characteristics of the subjects.

	Tai Chi (*n* = 14)	Control group (*n* = 14)	*t* or *χ* ^2^	*P*
	Mean ± SD	Mean ± SD		

Age (yr)	71.79 ± 3.38	69.57 ± 5.81	1.23	.23

	Number (%)	Number (%)		

Married				
Yes	14 (100)	12 (92)	1.19	.48
No	0 (0)	1 (8)		
Educational level				
Junior high	2 (15)	5 (36)	4.53	.10
High	3 (23)	6 (43)		
College	8 (62)	3 (21)		
Employed				
Yes	3 (21)	1 (7)	1.17	.60
No	11 (79)	13 (93)		
Monthly income (in ten thousand Won)				
<100	6 (44)	7 (50)	.89	.93
100–150	3 (21)	4 (29)		
151–200	2 (14)	1 (7)		
201–250	3 (21)	2 (14)		
Perceived health status				
Healthy	4 (29)	5 (36)	.60	1.00
Average	8 (57)	6 (43)		
Bad	2 (14)	3 (21)		
Surgical history for the prostate				
Yes	2 (14)	1 (7)	.37	.54
No	12 (86)	13 (93)		
Medication for the prostate				
Yes	6 (43)	8 (57)	.57	.71
No	8 (57)	6 (43)		
Exercise				
Yes	9 (64)	13 (93)	3.39	.17
No	5 (36)	1 (7)		

SD: standard deviation.

**Table 2:** Homogeneity tests of the outcomes between the groups.

Outcome	Tai chi (*n* = 14)	Control (*n* = 14)	*t*	*P*
IPSS	13.07 ± 7.96	16.36 ± 8.86	−1.03	0.31
QoL of BPH	43.64 ± 20.44	45.43 ± 19.78	−0.24	0.87
urination-related discomfort	12.43 ± 6.45	12.43 ± 6.66	0.001	1.00
Worry & concern	6.57 ± 3.86	6.14 ± 3.51	0.31	0.76
Interference with daily activities	11.57 ± 4.11	10.50 ± 4.27	0.68	0.51
Testosterone (ng/mL)	4.01 ± 1.05	3.65 ± 1.62	0.70	0.49
Insulin (*μ*U/mL)	11.30 ± 10.41	17.40 ± 29.05	−0.74	0.47
Blood glucose (mg/dL)	123.43 ± 26.12	128.21 ± 28.89	−0.46	0.65
QUICKI	0.35 ± 0.06	0.44 ± 0.41	−0.79	0.44

Values are expressed as mean ± standard deviation; IPSS: international prostate symptoms score; QoL of BPH: quality of life of benign prostate hyperplasia

QUICKI: quantitative insulin sensitivity check index.

**Table 3:** Effects of tai chi on IPSS, QoL, and biochemical outcomes.

Outcome	Tai chi (*n* = 14)			Control (*n* = 14)			*t *	*P*
	Before	After	After-Before	Before	After	After-Before		
IPSS	13.07 ± 7.96	8.93 ± 3.38	−4.14 ± 7.16	16.36 ± 8.86	17.07 ± 9.04	.71 ± 2.87	−2.36	0.03
QoL of BPH (Total)	43.64 ± 20.44	33.07 ± 12.38	−10.57 ± 14.76	45.43 ± 19.79	48.50 ± 19.51	3.07 ± 7.84	−3.06	0.005
QoL of BPH (subscale)								
urination-related discomfort	12.43 ± 6.45	10.00 ± 4.90	−2.43 ± 5.69	12.43 ± 6.66	13.29 ± 6.47	0.86 ± 2.21	−2.01	0.06
Worry & concern	6.57 ± 3.86	4.57 ± 3.69	−2.00 ± 2.11	6.14 ± 3.51	6.43 ± 3.39	.29 ± 2.79	−2.45	0.02
Interference with daily activities	11.57 ± 4.11	9.57 ± 4.11	−2.00 ± 3.23	10.50 ± 4.27	11.71 ± 5.59	1.21 ± 5.31	−1.94	0.06
Testosterone (ng/mL)	4.01 ± 1.05	6.06 ± 1.91	2.05 ± 1.65	3.65 ± 1.62	4.17 ± 1.56	0.52 ± 1.08	2.92	0.007
Insulin (*μ*U/mL)	11.30 ± 10.41	3.97 ± 3.39	−7.33 ± 8.66	17.40 ± 29.05	11.16 ± 19.62	−6.23 ± 12.28	−0.27	0.79
Blood glucose (mg/dL)	123.43 ± 26.12	114.07 ± 21.77	−9.36 ± 16.29	128.21 ± 28.89	119.43 ± 21.77	−8.79 ± 17.01	−0.09	0.93
QUICKI	0.35 ± 0.06	0.48 ± 0.31	0.13 ± 0.23	0.44 ± 0.41	0.38 ± 0.07	−0.06 ± 0.41	1.38	0.18

The value are expressed as mean ± standard deviation; IPSS: international prostate symptoms score; QoL of BPH: quality of life of benign prostate hyperplasia; QUICKI: quantitative insulin sensitivity check index.
